# Promoting mental health and well-being in schools: examining mindfulness, relaxation and strategies for safety and well-being in English primary and secondary schools—study protocol for a multi-school, cluster randomised controlled trial (INSPIRE)

**DOI:** 10.1186/s13063-023-07238-8

**Published:** 2023-03-23

**Authors:** Daniel Hayes, Anna Moore, Emily Stapley, Neil Humphrey, Rosie Mansfield, Joao Santos, Emma Ashworth, Praveetha Patalay, Eva-Maria Bonin, Sara Evans-Lacko, Bettina Moltrecht, Kirsty Nisbet, Emma Thornton, Aurelie Lange, Paul Stallard, Abigail Thompson, Jan Rasmus Boehnke, Jessica Deighton

**Affiliations:** 1 Evidence Based Practice Unit (EBPU), University College London and Anna Freud National Centre for Children and Families (AFNCCF), London, UK; 2 Institute of Education, The University of Manchester, Manchester, UK; 3 MRC Unit for Lifelong Health and Ageing and the Centre for Longitudinal Studies, University College London, London, UK; 4 Personal Social Services Research Unit, London School of Economics and Political Science, London, UK; 5 Care Policy and Evaluation Centre (CPEC), London School of Economics and Political Science, London, UK; 6 Manchester Institute of Education, The University of Manchester, Manchester, UK; 7 Department for Health, University of Bath, Bath, UK; 8 School of Nursing and Health Sciences, University of Dundee, Dundee, UK

**Keywords:** Adolescent, Young person, Children, Cluster randomised controlled trial, Mental health, Well-being, School-based

## Abstract

There are increasing rates of internalising difficulties, particularly anxiety and depression, being reported in children and young people in England. School-based universal prevention programmes are thought to be one way of helping tackle such difficulties. This paper describes an update to a four-arm cluster randomised controlled trial (http://www.isrctn.com/ISRCTN16386254), investigating the effectiveness of three different interventions when compared to usual provision, in English primary and secondary pupils. Due to the COVID-19 pandemic, the trial was put on hold and subsequently prolonged. Data collection will now run until 2024. The key changes to the trial outlined here include clarification of the inclusion and exclusion criteria, an amended timeline reflecting changes to the recruitment period of the trial due to the COVID-19 pandemic and clarification of the data that will be included in the statistical analysis, since the second wave of the trial was disrupted due to COVID-19.

**Trial registration** ISRCTN Registry ISRCTN16386254. Registered on 30 August 2018.

**Supplementary Information:**

The online version contains supplementary material available at 10.1186/s13063-023-07238-8.

## Introduction

This paper describes an update to a four-arm cluster randomised controlled trial (http://www.isrctn.com/ISRCTN16386254) [1], investigating the effectiveness of three different interventions when compared to usual provision, in English primary and secondary pupils. Due to the COVID-19 pandemic, the trial was put on hold and subsequently prolonged. Data collection will run until 2024.

## Inclusion/exclusion criteria

In addition to the original inclusion criteria for schools [1], schools are only eligible for participation in the trial if they sign a Memorandum of Understanding, data sharing agreement, and provide pupil lists to the research team. These criteria have been added to provide more clarity because they were already included in the process but had not been explicitly stated in the inclusion/exclusion criteria, so it was deemed necessary to be explicit that these steps are requirements.

## Timeline

Recruitment of schools for wave 1 opened in March 2018 and closed in September 2018. Schools recruited in wave 1 completed the baseline assessment in September to October 2018 (time 1), delivered the intervention from January to March 2019 and completed the follow-up assessment in May 2019 (3–6 months time point, time 2) and January to March 2020 (9–12 months time point, time 3).

Recruitment of schools for wave 2 opened in February 2019 and closed in June 2019. Schools recruited in wave 2 completed the baseline assessment in September to October 2019 (time 1) and delivered the intervention from January to the end of March 2020, with follow-up assessments due in May to June 2020 (time 2) and January to March 2021 (time 3). Due to the start of the COVID-19 pandemic in the UK in March 2020, delivery of the interventions could not be finalised, and the follow-up assessments due in May to June 2020 (time 2) could not be conducted (including the primary outcome). The follow-up assessment in January to March 2021 (time 3) was collected.

To achieve the required sample size, recruitment recommenced in January 2022 and closed in September 2022 (wave 3). Recruitment of schools for wave 3 opened in February 2022 and closed in September 2022. These schools will complete the baseline assessments in September to October 2022 (time 1), deliver the intervention from January to March 2023 and complete the follow-up assessments in May 2023 (time 2) and January to March 2024 (time 3). See the Supplementary material for an updated timeline.

Due to the challenges around data collection created by the COVID-19 pandemic, the following data will be available at the end of the trial:

- Wave 1: baseline (time 1), post-intervention (time 2) and 1-year follow-up (time 3)
- Wave 2: baseline (time 1) and 1-year follow-up (time 3)
- Wave 3: baseline (time 1), post-intervention (time 2) and 1-year follow-up (time 3)

## Statistical analysis of the primary outcome

As the primary outcomes for all trials remain unchanged and are measured at 3–6 months follow-up (time 2), only wave 1 and wave 3 data will be used for the primary outcome analysis. The original protocol proposed to estimate three mixed models, each comparing an active treatment with the control arm. We would like to clarify that these will be conducted separately for primary and secondary schools. These two contexts are significantly different in terms of the developmental stage of the children and also the educational setting, so it is deemed preferable to analyse them separately. The analytic strategy set out in the original protocol remains unchanged, with mixed models allowing for school-level clustering, control for baseline levels in the primary outcome and the minimisation variables. As outlined in the original protocol, the statistical power of the strategy allows for minimally detectable effect sizes (MDES) of MDES = .186 in primary and MDES = .173 in secondary schools. We will conduct sensitivity analyses to test for the differences between waves. As stated in the original protocol, a detailed statistical analysis plan will be written prospectively and made publicly available.

### Roles and responsibilities

As this is an ongoing trial, there have been various people undertaking similar roles at different stages, outlined below. JD is the principal investigator. NH is the implementation lead. DH was the trials manager for waves 1 and 2. AT is the trials manager for wave 3. AL supported in between trial managers. JS was the data manager for waves 1 and 2, with ongoing support for wave 3. ET is the data manager for wave 3. ES is the qualitative lead. AM was the research officer/school liaison lead for waves 1 and 2, with ongoing support for wave 3. KN is the research officer/school liaison lead for wave 3. RM and EA were research assistants. JB is the trials statistician. PP provides expertise in measures and statistical analysis. BM provides expertise in evaluating mindfulness practices. EB was the health economist for waves 1 and 2. SL is the health economist for wave 3.

## Supplementary Information

**Additional file 1.**

## Data Availability

An anonymised quantitative dataset generated and/or analysed during the current study will be available in 2024 once the study has finished. A decision regarding storage location is yet to be finalised. Please contact the principal investigator or trials manager for further information.

## Abbreviations

AEs: Adverse events
DMC: Data monitoring committee
GDPR: General Data Protection Regulation
MDES: Minimally detectable effect sizes
SAEs: Serious adverse events
UCL: University College London
