# Supplementary material for: Promoting mental health and well-being in schools: examining mindfulness, relaxation and strategies for safety and well-being in English primary and secondary schools—study protocol for a multi-school, cluster randomised controlled trial (INSPIRE)
Source: Trials. 2023 Mar 23;24:220. doi: 10.1186/s13063-023-07238-8 (PMC10034911; doi:10.1186/s13063-023-07238-8)
Supplement: Supplementary file 1 [file 13063_2023_7238_MOESM1_ESM.odt]

Timeline

Main trial 

| Task | Timeline |
| --- | --- |
| Wave 1 |
| Recruitment of schools | Mar 18 – Sept 18 |
| Usual provision surveys (Time 1) | Jun 18 – Oct 18 |
| Recruitment of pupils in relevant classes and year groups (via information sheets and consent forms) | Jul 18 – Oct 18 |
| Baseline measures (Time 1; pupil and teacher) | Sep 18 – Oct 18 |
| Randomisation | Oct 18 |
| Intervention delivery | Jan 19 – Apr 19 |
| Case study visits (Time 1; observations and interviews) | Mar 19 - Apr 19 |
| Implementation surveys | April 19 – May 19 |
| Follow up measures (Time 2; teacher and pupil) | May 19 -Jun 19 |
| Finance survey | May 19 – Jul 19 |
| Follow up measures (Time 3; teacher) | Jan 20 – Feb 20 |
| Follow up measures (Time 3; pupil) | Jan 20 – Feb 20 |
| Usual provision surveys (Time 2) | Nov 19 |
| Case study visits (Time 2; observations and interviews) | Jan 20 – Feb 20 |
| Wave 2 |
| Recruitment of schools | Feb 19 – Jun 19 |
| Usual provision surveys (Time 1) | May 19 – Oct 19 |
| Recruitment of pupils in relevant classes and year groups (via information sheets and consent forms) | Jul 19 – Oct 19 |
| Baseline measures (Time 1; pupil and teacher) | Sep 19 – Oct 19 |
| Randomisation | Oct 19 |
| Intervention delivery | Jan 20 – Mar 20 |
| Implementation surveys | April 20 – May 20 |
| Follow up measures (Time 2; teacher and pupil) | May 20 -Jun 20 |
| Finance survey | May 20 – Jul 20 |
| Follow up measures (Time 3; teacher) | Jan 21 – Mar 21 |
| Follow up measures (Time 3; pupil) | Jan 21 – Mar 21 |
| Usual provision surveys (Time 2) | Nov 20 |
| Data analysis cleaning and preparation for final analysis across Waves 1 and 2 | Mar 21 |
| Wave 3 |
| Recruitment of schools | Feb 22 – Sept 22 |
| Usual provision surveys (Time 1) | May 22 – Oct 22 |
| Recruitment of pupils in relevant classes and year groups (via information sheets and consent forms) | Jul 22 – Oct 22 |
| Baseline measures (Time 1; pupil and teacher) | Sep 22 – Oct 22 |
| Randomisation | Oct 22 |
| Intervention delivery | Jan 23 – Mar 23 |
| Implementation surveys | April 23 – May 23 |
| Follow up measures (Time 2; teacher and pupil) | May 23 -Jun 23 |
| Finance survey | May 23 – Jul 23 |
| Follow up measures (Time 3; teacher) | Jan 24 – Mar 24 |
| Follow up measures (Time 3; pupil) | Jan 24 – Feb 24 |
| Usual provision surveys (Time 2) | Nov 23 |
| Data analysis cleaning and preparation for final analysis across Waves 1, 2 and 3 | Mar 24 |
| Data analysis of Wave 1, Wave 2 and Wave 3 data | Apr 24 – May 24 |
